# Retraction: Fibrillin-1, induced by Aurora-A but inhibited by BRCA2, promotes ovarian cancer metastasis

**DOI:** 10.18632/oncotarget.28860

**Published:** 2026-04-24

**Authors:** Ziliang Wang, Yang Liu, Lili Lu, Lina Yang, Sheng Yin, Yan Wang, Zihao Qi, Jiao Meng, Rongyu Zang, Gong Yang

**Affiliations:** ^1^Cancer Institute, Fudan University Shanghai Cancer Center, Shanghai 200032, China; ^2^Department of Gynecological Oncology, Fudan University Shanghai Cancer Center, Shanghai 200032, China; ^3^Department of Oncology, Shanghai Medical College, Fudan University, Shanghai 200032, China; ^4^Department of Biology, Life and Environment Science College, Shanghai Normal University, Shanghai 200023, China; ^5^Central Laboratory, the Fifth People’s Hospital of Shanghai Fudan University, Shanghai 200240, China; ^*^These authors have contributed equally to this work

**This article has been retracted:** This decision follows an investigation by Oncotarget journal regarding a correction request from the corresponding author, Dr. Gong Yang. However, our image forensics analysis revealed multiple instances of internal duplications and overlaps.

Specifically:

- Figure 1B: The western blot image for OVCA429/MMP13 is a duplicate of OVCA420 E-cadherin. Furthermore, the β-actin blots for both cell lines are duplicates of the β-actin blots in Figure 3A.
- Figure 2A: The OVCA429/control siRNA migration assay image overlaps with the invasion control. The OVCA420 invasion assay contains two overlapping images for Aur A cDNA and BRCA2 shRNA transfected cells. Additionally, the OVCA420/Aur A cDNA and OVCA420/BRCA2 shRNA invasion images overlap with the OVCA429/siRNA Control migration image in Figure 3E.
- Figure 3D: The FBN1-/E-cadherin band of the OVCA429/BRCA2 cDNA western blot is a duplicate of the FBN+/β-actin band.
- Figure 3E: In the transwell assay, duplicate images were found between OVCA429/FBN1 siRNA (migration) and OVCA420/Aur A cDNA/siRNA Control (invasion). Overlaps also occurred between migration assay images for OVCA420/Aur A cDNA/FBN1 siRNA and OVCA420/BRCA2 shRNA/FBN1 siRNA treated cells.
- Figure 4B: The western blots for β-actin were duplicated between OVCA429 and OVCA420/Aur A cDNA.

The corrected versions of Figures 2, 3, and 4 initially provided by the authors were not supported by the original data. Furthermore, the authors did not respond to multiple requests from the Scientific Integrity office for explanations and raw data. Given the volume of inconsistencies and the absence of supporting materials, the Editorial decision has been made to retract the article. The authors have been notified of this final decision but have not responded.

Original article: Oncotarget. 2015; 6:6670–6683. 6670-6683. https://doi.org/10.18632/oncotarget.3118

